# Chromosomal rearrangements, phenotypic variation and modularity: a case study from a contact zone between house mouse Robertsonian races in Central Italy

**DOI:** 10.1002/ece3.1912

**Published:** 2016-01-30

**Authors:** Paolo Franchini, Paolo Colangelo, Axel Meyer, Carmelo Fruciano

**Affiliations:** ^1^ Department of BiologyLehrstuhl für Zoologie und EvolutionsbiologieUniversity of KonstanzUniversitätsstraße 1078457KonstanzGermany; ^2^ Dipartimento di Biologia e Biotecnologie “Charles Darwin”Universitá di Roma “La Sapienza”via Borelli 5000161RomaItaly; ^3^ CNRIstituto per lo Studio degli EcosistemiVerbaniaItalia; ^4^ School of EarthEnvironmental and Biological SciencesQueensland University of TechnologyGardens Point4000BrisbaneAustralia

**Keywords:** Chromosomal speciation, geometric morphometrics, house mouse, modularity, phenotypic variation, Robertsonian fusions

## Abstract

The Western European house mouse, *Mus musculus domesticus*, is well‐known for the high frequency of Robertsonian fusions that have rapidly produced more than 50 karyotipic races, making it an ideal model for studying the mechanisms of chromosomal speciation. The mouse mandible is one of the traits studied most intensively to investigate the effect of Robertsonian fusions on phenotypic variation within and between populations. This complex bone structure has also been widely used to study the level of integration between different morphogenetic units. Here, with the aim of testing the effect of different karyotypic assets on the morphology of the mouse mandible and on its level of modularity, we performed morphometric analyses of mice from a contact area between two highly metacentric races in Central Italy. We found no difference in size, while the mandible shape was found to be different between the two Robertsonian races, even after accounting for the genetic relationships among individuals and geographic proximity. Our results support the existence of two modules that indicate a certain degree of evolutionary independence, but no difference in the strength of modularity between chromosomal races. Moreover, the ascending ramus showed more pronounced interpopulation/race phenotypic differences than the alveolar region, an effect that could be associated to their different polygenic architecture. This study suggests that chromosomal rearrangements play a role in the house mouse phenotypic divergence, and that the two modules of the mouse mandible are differentially affected by environmental factors and genetic makeup.

## Introduction

The causative role of chromosomal rearrangements in speciation is a prominent issue in evolutionary biology (e.g., Rieseberg 2001; Faria and Navarro 2010). Despite the accumulation of empirical evidence showing that chromosomal rearrangements could contribute to reproductive isolation, the debate on whether the karyotypic differences promote species divergence or these arise after the completion of the speciation process is still open (Coyne and Orr 2004).

Among the different classes of chromosomal rearrangements (e.g., translocations, inversions, fusions), several reasons lead to hypothesize an active role of Robertsonian (Rb) fusions in animal speciation. This mechanism produces Rb chromosomes, metacentrics resulting from the fusion of two acrocentric chromosomes at their centromere, translocations that involve the so called centric fusions or fissions between chromosome arms, causing a change in diploid number, but not chromosome arm number. For this reason, these large‐scale karyotipic reorganizations poorly alter the genomic content of a species (Garagna et al. 2001), but they can affect its gene architecture. It has been shown, indeed, how translocated chromosomes might experience a reduced recombination rate (especially in pericentromeric regions) due to physical impedance to form chiasma during meiosis (Bidau et al. 2001; Castiglia and Capanna 2002; Dumas and Britton‐Davidian 2002; Franchini et al. 2010), thus altering the linkage between alleles of *loci* that influence different traits. For the same mechanism, the reduced meiotic recombination in specific regions of a chromosome can facilitate the fixation of allelic variants with different pleiotropic effect in a population, allowing the expression of specific phenotypic traits.

In this context, it is perhaps important to notice how traits of organisms do not vary independently, but are integrated with each other, reflecting coordination in development, function and evolution (Klingenberg 2010). Traits that are relatively independent from each other are often called modules (Klingenberg et al. 2003, 2004; Wagner et al. 2007). Modularity has been the subject of intensive research in different systems from macroevolutionary studies across distantly related taxa (Sanger et al. 2012; Goswami et al. 2014) to intraspecific analyses (Drake and Klingenberg 2010; Muñoz‐Muñoz et al. 2011) in order to address questions in developmental and evolutionary biology. From an evolutionary perspective, indeed, if different characters are able to vary independently, selection will be able to optimize each character separately. For this reason, the concept of modularity has been linked to evolvability, the ability of a biological unit to respond to a selective challenge (Hansen 2003).

One of the most intensively studied traits to investigate the effect of Robertsonian fusions in producing intra‐ and interpopulations phenotypic differences is the mouse mandible (Corti and Rohlf 2001; Sans‐Fuentes et al. 2009; Muñoz‐Muñoz et al. 2011; Martinez‐Vargas et al. 2014). Moreover, this trait has been recently used to study the integration between different morphological units, thus their covariation under standard and perturbed developmental conditions (e.g., occurrence of Robertsonian chromosomes) (Muñoz‐Muñoz et al. 2011; Martinez‐Vargas et al. 2014).

**Figure 1:**
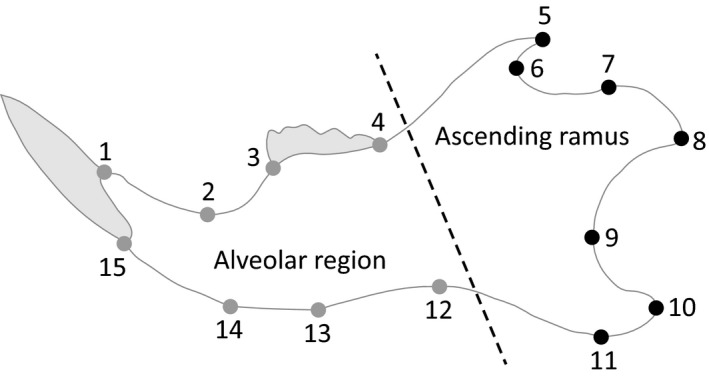
Lingual view of the left mandible of the house mouse showing the location of the 15 selected landmarks. The dashed line separates the two main modules of the mandible, the alveolar region and the ascending ramus.

One of the advantages of the mouse mandible in this context is that it is a model system to study the development and evolution of complex morphological structures (Klingenberg 2010). This bone structure is made of six different units that differ in their embryological origin, time and rates of differentiation (Atchley and Hall 1991). The six parts are grouped in two main functional units, the alveolar region (the distal region of the mouse mandible that houses the teeth) and the ascending ramus (the region that connects the mandible to the skull and in which the masticatory muscles are connected) (Fig. 1), (Leamy 1993; Klingenberg et al. 2003). Quantitative trait *loci* (QTL) analyses have shown a certain degree of genetic independence between these two modules (Ehrich et al. 2003; Klingenberg et al. 2004).

Rb fusions are particularly frequent in the Western European house mouse, *Mus musculus domesticus* that, thanks to the frequent occurrence and fast fixation of Rb chromosomes, has rapidly become a model organism to understand how chromosomal rearrangements might play a role in the establishment of reproductive isolation and affecting phenotypic traits. More than 50 chromosome races (populations in which Rb chromosomes are fixed in homozygosis) have been discovered so far across the distribution area of *M. m. domesticus* with a diploid number ranging from 40, the standard “all‐acrocentrics” situation typical of the genus *Mus*, to 22, the karyotype showing the highest level of Rb fusions (Sage et al. 1993; Pialek et al. 2005).

**Figure 2:**
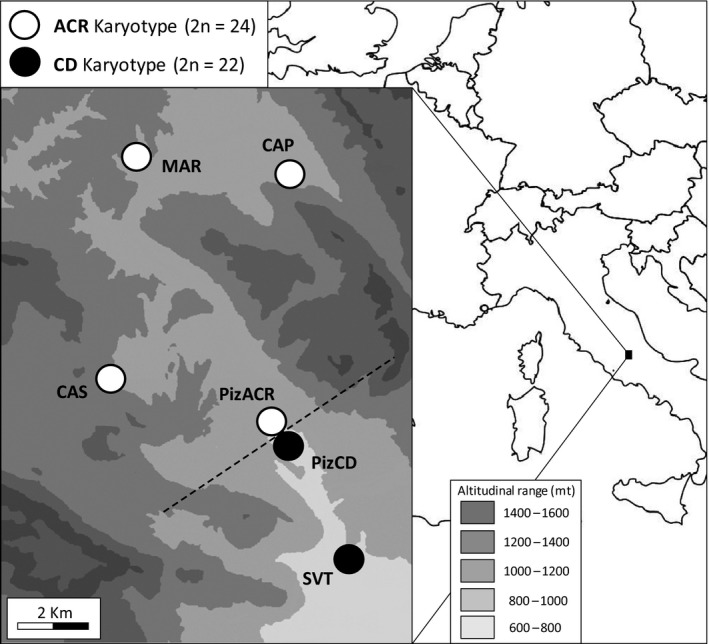
Map of the contact area between the CD and the ACR Robertsonian races. The six source populations of the mice are shown.

Here, we investigate the impact of chromosomal rearrangements in altering the mandible morphology and its modularity in a well‐known contact area between two chromosomal races in Central Italy (Fig. 2). Both races, Ancarano (ACR, somatic number 2*n* = 24) and Cittaducale (CD, 2*n* = 22), are characterized by a high level of metacentric chromosomes. As recently highlighted by cytogenetic and molecular genetic studies (Castiglia et al. 2002; Franchini et al. 2008), these two races are in advanced stages of the speciation continuum, as their genetic divergence is facilitated by the high level of infertility of F_1_ hybrids (consequence of the karyotipic incompatibility between the two races), and potentially with further divergence promoted by premating reinforcement (unpublished data).

The aim of this work is twofold. Firstly, we wanted to test for the effect of different karyotypic assets on the morphology of the mouse mandible. These two chromosomal races have specific geographical arrangements and patterns of reduction of gene flow which might cause the observed differences between races. For this reason, rather than simply testing for phenotypic differences between the two chromosomal races, we also explicitly test and account for these factors. Secondly, we wanted to investigate whether a pattern of modularity is supported in the mandible of these two races, whether the levels of modularity are the same between them and how the morphology of the two putative modules of the mandible responded to different potential sources of variation (i.e., environmental factors varying in geographic space and genetic perturbations driven by the deeply different karyotypic structure of the two races).

## Material and Methods

### Morphometric data collection

A sample of 84 mice was live‐trapped between June 1998 and March 2000 in Central Italy, along the Aterno River. After collection, the karyotype of each individual was characterized using G‐banding techniques and assigned to the ACR (66 specimens) or the CD race (18 mice) (Castiglia et al. 2002). The mice were successfully collected in 14 sampling localities where no co‐occurrence of the two races was found, not even in the village in which the races come in contact (Fig. 1; Table S1). On the basis of their geographical distances and in considerations on their habitat continuity (relevant parameters for a commensal species with a limited dispersal capability), in a previous study the 14 sampling sites were pooled in six populations in order to assess the genetic structure of the system and the signature of hybridization between the two races using microsatellite markers (Franchini et al. 2008). In the present study, we use the same grouping in six populations for the analyses which require an *a priori* definition of groups. At the time of collection, specimens were also weighted so that we could use weight as a measure of body size when comparing mandible size (for analyses of mandible shape we used mandible centroid size).

### Morphometric data collection and analyses

The left and right mandible of each specimen were separated at the mandibular symphysis and cleaned by Dermestid beetles. Being the one with the highest number of intact samples in the entire dataset, we used the left mandible to assess its size and shape variation in the 84 specimens. The mandible was placed flat on the dorsal side and the lingual side was photographed with a Nikon F100 camera equipped with a 105 mm macro lens (Nikon Inc., Tokyo, Japan). The mouse mandible is a nearly flat bone, so we assumed that the two‐dimensional representation provided by a photograph allows for a good approximation of its shape (Cardini 2014). Fifteen landmarks were collected using tpsDig2 (Rohlf 2008) as shown in Fig. 1. The configurations of points were then subjected to generalized Procrustes analysis (Rohlf and Slice 1990) and the resulting shape variables were used in subsequent analyses. We also computed mandible centroid size. Centroid size was used as a measure of mandible size when testing for mandible size variation between races using linear models with and without body weight as covariate (i.e., controlling for allometry in the latter case).

We tested for the influence of allometry on mandible shape variation by performing a multivariate regression of shape on centroid size as, obviously, allometric variation can obscure biologically relevant patterns of variation between races or, conversely, produce artifictual patterns of variation between groups.

Then, we fitted multiple linear models testing for the interactions race × centroid size and population × centroid size. We tested these interactions both in full factorial models including the main effects and in models with only centroid size and the relevant. There was no instance of a significant interaction term so in all the subsequent analyses we only tested for the main effects.

To test for a difference in shape between races we used two approaches. The first was performing in STATISTICA (StatSoft Inc) a nested MANCOVA using centroid size as covariate (to control for allometry) and population as a categorical factor nested in the chromosomal race categorical factor. The second consisted in using MorphoJ (Klingenberg 2011) to perform a regression of shape variables on centroid size and then using the regression residuals to test for differences between the two chromosomal races using the permutational procedure based on Procrustes distances implemented in MorphoJ (10,000 permutations). A discriminant analysis with leave‐one‐out cross‐validation was also performed on these regression residuals and used to identify shape differences between the two chromosomal races. A between‐group principal component analysis (Boulesteix 2005; Mitteroecker and Bookstein 2011; Franchini et al. 2014; Fruciano et al. 2014; Schmieder et al. 2015) based on population means was used as an exploratory tool to visualize the degree of overlap among populations.

We, then, tested for the relative contribution of genetic similarity and race. In fact, the two chromosomal races have experienced a reduction of gene flow (Franchini et al. 2008), so any phenotypic difference between the races might have arisen as a consequence of such reproductive isolation. However, if there is a significant difference between chromosomal races even after controlling for neutral genetic distances, an additional factor (such as the karyotype itself) must be invoked to explain any observed difference. First, based on the residuals of the regression of shape variables on centroid size (i.e., controlling for allometry) we computed in tpsSmall (Rohlf 2015) the pairwise tangent Procrustes distances between the 78 individuals for which microsatellite data was available. We then computed the correlation of this matrix of morphometric distances with a matrix of pairwise Nei distances (Nei et al. 1983; Takezaki and Nei 1996) based on microsatellite frequencies and tested its significance with a Mantel test (Mantel 1967) in NTSYSpc (Rohlf 2005). To test for the association between karyotype and morphology while controlling for genetic similarity, we created another among‐individual dissimilarity matrix – which we term a “matrix of karyotypic distances” – containing zero if two individuals had the same karyotype and one if they had different karyotypes. We then performed a partial Mantel test (Smouse et al. 1986) of the correlation between the matrix of karyotypic distances and the matrix of morphometric distances, while controlling for the matrix of genetic distances. We also approached this question using a model‐based approach. To this aim, we performed a principal coordinate analysis on the pairwise Nei distances retaining the scores along the first 19 principal coordinates (i.e., all the principal coordinates accounting for at least one percent of variance). We then fitted two linear models: a full model in which shape (dependent variables, already corrected for allometry) was a function of both chromosomal race and principal coordinate scores and a reduced model in which shape was a function of just the principal coordinate scores based on genetic data. Finally, to compare the two models we used the *advanced.procD.lm* function of the R package *geomorph* (Adams and Otárola‐Castillo 2013) which employs a residual randomization permutation procedure (Collyer et al. 2015) for hypothesis testing (1000 permutations).

Furthermore, we investigated the spatial variation in mandible morphology using spatially explicit methods. These are statistical methods that incorporate explicitly the spatial component and they have been used only in a limited number of geometric morphometric studies (Cardini et al. 2007; Fruciano et al. 2011). The rationale for using such methods in this study is that the populations studied here have a specific spatial arrangement and patterns of variation between the two races might arise as a consequence of this (i.e., because of geographic distance or environmental factors correlated with geography). It is, therefore, important to first test for phenotypic variation in geographic space and then, if such variation is present, account for it when comparing the two races. In particular, here we used a Mantel test (Mantel 1967) to test for the correlation of geographical and morphometric distances (these were tangent Procrustes distances obtained after removing the allometric component). We also used bearing analysis (Falsetti and Sokal 1993) to identify clines of shape variation in geographic space. Bearing analysis – introduced in genetics (Falsetti and Sokal 1993) and later on applied to geometric morphometric data (Fruciano et al. 2011) – consists in a procedure where the correlation between data distances (in this case morphometric distances) and geographic distances weighted relative to a specific direction in geographic space is computed and tested with a Mantel test. Being the geographical distances weighted relative to a specific direction, a significant correlation implies a significant trend or cline in that direction. Here, we did not have any *a priori* hypothesis of a clinal direction so we decided to use 36 different directions, 10° apart from each other (effectively covering 360°). Obviously, any geographical pattern of shape variation might be the consequence of the variation in geographic space of the genetic makeup and/or of the spatial arrangement of the two chromosomal races. To control for this, we further used partial Mantel tests to assess the correlation between morphometric and geographic pairwise distances among individuals while accounting for the effect of genetic and chromosomal distances. Perhaps most importantly, we also performed a partial Mantel test to test the significance of the correlation between the matrix of karyotypic distances and the matrix of morphometric distances while controlling for geographic distances. If such correlation is significant, spatial variation alone cannot explain differences between chromosomal races.

Considering that the mouse mandible is often used in studies of modularity and integration, we set out to investigate if different karyotypes had different levels of modularity (Fruciano et al. 2013). To this aim, we first used – on the dataset corrected for allometry – the method (Klingenberg 2009) to test hypotheses of modularity implemented in MorphoJ both on the full dataset and on each chromosomal races separately. Then, we applied two recently developed approaches (Fruciano et al. 2013) to test for differences in the level of modularity between groups. In particular, we obtained estimates of the Escoufier RV coefficient (Escoufier 1973) for each chromosomal race rarefied to the smallest sample size and a permutation test for the null hypothesis of no difference in levels of modularity between the two chromosomal races (Fruciano et al. 2013).

Furthermore, we also removed the allometric component through regression on centroid size for each module separately and then performed a series of analyses on each module. In particular, using data for one module at a time we tested for difference in mean shape between chromosomal races and we performed tests of association between morphological and genetic/karyotypic/geographic distances. In fact, we performed a partial Mantel test for the association between morphometric and karyotypic distances while accounting for genetic distances, computed as chord distance (Cavalli‐Sforza and Edwards 1967). We also performed Mantel tests for the association of morphometric and genetic/geographic distances on each module separately.

## Results

Our analysis of centroid size did not reveal any significant difference between chromosomal races (*F* = 0.11, *P* = 0.74). When weight was introduced as covariate in the model (thus correcting for allometry), it was the only significant term, but mandible centroid size was still not significant. This indicates that there is no difference in mandible size between races.

**Figure 3:**
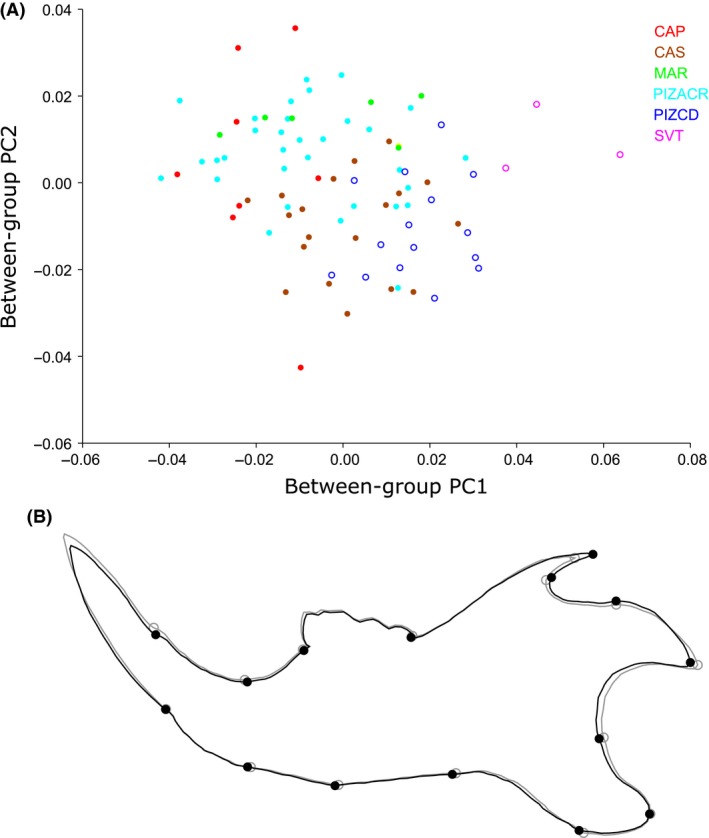
(A) Scatterplot of the scores along the first two between‐group principal components computed using populations as groups. Empty circles: CD karyotype; filled circles: ACR karyotype. (B) Difference in mean mandible shape between the two chromosomal races. The light gray line represents the shape for the ACR race, the black line the shape for the CD race.

The regression of mandible shape on centroid size was significant (*P* = 0.0005) but it accounted for a small proportion (4.3%) of the variation in shape. We found a significant difference in shape between the two chromosomal races with both the approaches we used to test for this effect. In particular, in the nested MANCOVA, both population (Wilks Lambda 0.061; df = 104; *P* = 0.000006) and race (Wilks Lambda 0.36; df = 26; *P* = 0.00005) were highly significant terms. The permutation test for the null hypothesis of no difference in mean shape between the two chromosomal races was also highly significant (*P* < 0.0001) and the discriminant analysis showed a relatively high cross‐validated correct classification rate (76.19%). The exploratory plot (Fig. 3) of the scores along the first two between‐group principal components (accounting for 40.25% and 22.65% of the variation in the full allometry‐corrected dataset, respectively) shows a certain degree of overlap among populations and, to a lesser extent, chromosomal races.

The correlation between genetic and morphometric distances is relatively low (*r* = 0.15) but highly significant (*P* = 0.0003). The partial Mantel test revealed a relatively low, but significant, correlation (*r* = 0.18, *P* = 0.0012) between karyotypic and morphometric distances when controlling for genetic distances. The modeling approach we used to assess the relative contribution of genetic makeup and karyotype showed that the model incorporating both terms was significantly better than the one accounting only for genetic makeup (*F* = 1.6412, *Z* = 2.0052 *P* = 0.01).

The analyses of variation in geographic space revealed a significant correlation between morphometric and geographic distances (*r* = 0.11, *P* = 0.014). Perhaps most importantly, the bearing analysis was significant for a range of angles (60–135°), with the highest correlation at 100° (*r* = 0.21, *P* < 0.001), corresponding to an approximate direction North‐West to South‐East. This is the same direction along which the two chromosomal races are separated. Partial Mantel tests of the correlation between geographic and morphometric distances were significant both when accounting for genetic distances (*r* = 0.11, *P* = 0.025) and when accounting for karyotypic differences (*r* = 0.15, *P* = 0.006), thus suggesting that these two factors cannot be the only explanation for the geographical variation in mandible shape. Conversely, we also found a significant correlation (*r* = 0.2, *P* = 0.0008) between karyotipic distances and morphometric distances while controlling for geographic distances.

When using Klingenberg's method for the analysis of modularity, the proportion of random partitions with an Escoufier RV coefficient higher than the RV coefficient observed in the datasets (normally interpreted in the same way as a *P* value) was always small (full dataset = 0.04; CD = 0.007; ACR = 0.005). However, the RV coefficient rarefied at the same sample size was remarkably similar between the two chromosomal races (CD = 0.44; ACR = 0.42) and the test for the difference in RV coefficient between the two races was not significant (*P* = 0.758). This suggests that there is a significant modularity in mandible of these two chromosomal races but the “strength” of this modularity is similar across the two races.

Both permutation tests for difference in multivariate mean between chromosomal races performed on each module were significant (anterior module, Procrustes distance 0.025 *P* = 0.018; posterior module, Procrustes distance 0.059 *P* < 0.001).

When performing tests of association on the anterior module, we found a significant association between geographic and morphometric distances (*r* = 0.1, *P* = 0.043). However, we did not detect any significant association between morphometric and karyotypic distances when controlling for genetic distances (*r* = 0.05, *P* = 0.23) nor between morphometric and genetic distances (*r* = 0.02, *P* = 0.28).

On the contrary, when analysing the posterior module we found exactly the opposite pattern. That is, we did not find a significant association of geographic and morphometric distances (*r* = 0.07, *P* = 0.064) but significant association between morphometric and karyotypic distances when controlling for genetic distances (*r* = 0.22, *P* = 0.0002) and significant correlation between morphometric and genetic distances (*r* = 0.14, *P* = 0.0004).

## Discussion

Are chromosomal rearrangements active drivers of species divergence or do the observed karyotypic differences arise and become fixed in populations after the speciation process? Despite the intensive research that has been conducted so far to address this question, it remains a hotly debated issue and a general consensus has yet to be reached (Coyne and Orr 2004). The Western European house mouse, *Mus musculus domesticus*, offers a unique case to address these questions as Robertsonian translocations are extremely common in this subspecies. In particular, hybrid zones between Robertsonian mice are powerful systems to study how large‐scale chromosomal translocations can modify species’ phenotype, thus altering their evolutionary potential and ultimately contributing to reproductive isolation (even in the presence of the homogenizing effect of gene flow).

Here, we analysed morphological variation in the house mouse mandible in a contact zone between two Robertsonian races in Central Italy. Both races are characterized by a karyotype with a high number of metacentrics (CD race: 2*n* = 22; ACR race: 2*n* = 24), but they do not share any fusion involving the same chromosomal arms. Using centroid size (CS) as a measure of size, we did not find any particular pattern of variation that can be associated to the different race‐specific karyotype. Previous studies targeting Robersonian systems in Northern Italy and in Southern Spain showed that mice harboring standard karyotype and mice with a high diploid number (few centric fusions) have generally larger mandibles that those highly metacentric (Corti and Rohlf 2001; Muñoz‐Muñoz et al. 2011; Martinez‐Vargas et al. 2014). The focal races studied here have both a karyotype with a reduced number of chromosomes, not allowing us to test the size difference potentially promoted by a consistent difference in their diploid number.

While the mandible size was found to be similar in the two Rb races, we showed how mice harboring different Robertsonian chromosomes have distinguishable mandible shape. Importantly, we decided to explicitly test and control for two factors that could have produced patterns of variation between chromosomal races, namely reduction of gene flow (as measured by neutral genetic distances) and geographic position. While these factors might have a role in producing differences between chromosomal races, we demonstrate that chromosomal races have a different mandible shape even when controlling for these factors. Previous studies tested for a pattern of isolation‐by‐distance (IBS), a model that can be heavily biased in a commensal species where passive transport by humans could be the main factor affecting the house mouse distribution. IBS, in fact, was not detected in this study system when the correlation of genetic and geographic distances was assessed with a Mantel test (Franchini et al. 2008). The mice used for this survey were previously genotyped at mitochondrial (Castiglia et al. 2002) and microsatellite markers (Franchini et al. 2008). Those studies highlighted an advanced state of reproductive isolation between the races (expected by their highly divergent Robertonian karyotipic structures) and gave us the opportunity, especially using the population genetics parameters inferred by microsatellites, to correlate the genetic and morphological distances of the mice datasets, giving us more power to detect the karyotipic‐induced shape variation. In fact, our analyses show that, when using the whole landmark configuration, the genetic and morphometric distances are significantly correlated, confirming the genetic bases underlying the mandible shape (Ehrich et al. 2003; Klingenberg et al. 2004).

Moreover, we confirmed the evolutionary independence of the two regions of the mouse mandible for both races, a pattern that has been recently observed in Robertonian mice suggesting that Robertsonian translocations do not alter the modularity of the mouse mandible (Sans‐Fuentes et al. 2009; Muñoz‐Muñoz et al. 2011; Martinez‐Vargas et al. 2014). As shown by QTL mapping (Ehrich et al. 2003; Klingenberg et al. 2004), the mouse mandible size and shape are characterized by a highly polygenic architecture. The reduced meiotic recombination rate experienced by metacentric chromosomes (Klingenberg 2010) could have linked genes underlying the shape of the two mandible modules, thus increasing their level of integration. Further, not only physical linkage, but also the fixation of alleles with pleiotropic effect could have been promoted by the reduced recombination rate in certain chromosome regions. This hypothesis is supported by studies that reported a negative correlation between the number of metacentric chromosomes and the level of modularity of the mouse mandible (Muñoz‐Muñoz et al. 2011; Martinez‐Vargas et al. 2014). Following these evidences, in the present study we could have expected a higher level of modularity for the ACR race (the CD race has a diploid number of 22, the lowest found in the house mouse, where only the autosome 19 is not fuse to form a metacentric chromosome). However, the rarefied RV values of the two races are comparable and their difference not significant, not allowing us to reject the null hypothesis of similar modularity level between the focal races.

Interestingly, when we analysed phenotypic variation at the two modules independently, the correlation between the ascending ramus shape and the genetic distance of the specimens was higher and significant, while a lower and not significant value was estimated for the alveolar region. The alveolar region, the region housing the teeth, is potentially more influenced by environmental factors, as for example the diet. The house mouse is a species predominantly commensal to humans and, in the specific case of this study, mice were collected in similar farming habitations. A comparable diet regime could explain why a plastic response might have contributed to shape the alveolar region, partially hiding the genetically induced source of morphological variation. Phenotypic adaptive plasticity has been reported for the mouse mandible (Renaud and Auffray 2010; Anderson et al. 2014), with studies highlighting that both modules are influenced by a change in diet. However, Robertsonian karyoptipic configurations have not been targeted in such surveys and the different pattern we observed in the present study could suggest that a nongenetic phenotypic response in the alveolar region might be increased in metacentric races. The ascending ramus contains traits that are highly heritable and also controlled by a larger number of QTLs than those underlying the alveolar region (Ehrich et al. 2003; Klingenberg et al. 2004). It has been shown how Robertsonian chromosomes could reduce recombination rate in specific areas of fused chromosomes, thus reducing gene exchange in these regions (Bidau et al. 2001; Rieseberg 2001; Ayala and Coluzzi 2005; Faria and Navarro 2010; Franchini et al. 2010). This “linkage‐dependent” gene flow could statistically have higher chances to affect alleles controlling the ascending ramus (as they are more numerous) than the lower number of alleles involved in the alveolar process shape, this resulting in an increased divergence of the latter associated to the karyotypic configuration.

One of the main aims of this study was to test the effect of the different karyotypic configurations in altering the morphology of the mandible. The genetic data available allowed us to disentangle the source of morphological variation due to gene flow to that, if any, due to a specific karyotype composed by different metacentric fusions. Using a nested general linear model, the null hypothesis of similar shape of the mandible for the two races was rejected, showing that karyotype with different metacentric combinations might differentially alter its morphology. The comparison of average shape performed on each module independently showed the same general pattern emerged from the analysis of the whole mandible (i.e., a significant difference between races). However, the difference between races (as measured by the Procrustes distances between race means) was more pronounced when analysing the ascending ramus, suggesting that either phenotypic plasticity and stochastic sources of variation are affecting more the anterior module or that the effect of chromosomal rearrangements on mandible shape is more pronounced in the posterior module, possibly because of their higher number of QTL *loci* (see above).

Our study opens new doors for future integrated studies on determining how Robertsonian translocations can alter phenotypic traits and ultimately contribute to the reproductive isolation between populations. As we focused here on two Robertsonian races with an extremely reduced number of chromosomes, the obvious next step is to estimate morphological variation and modularity in other races where population genetic resources are available, preferably focusing on contact areas between races with different karyotypic structures. Moreover, the use of genome‐wide techniques (e.g., SNP‐chip or RAD‐Seq) will allow us to confirm the patterns we observed here and to identify the genomic regions contributing to shape variation between chromosomal races (as the location of the main QTL for mandible shape is known). These would be fundamental steps to shed new light on the contribution of linkage and pleiotropy in altering the morphology of the mandible and other phenotypic traits in Roberstonian systems, allowing the populations to evolve along different trajectories.

## Conflict of Interest

None declared.

## Supporting information


**Table S1.** Information of the 84 individuals used in this study.
